# Factors Influencing Press Ganey Ambulatory Surgery Scores in Patients Undergoing Upper Extremity Procedures

**DOI:** 10.5435/JAAOSGlobal-D-20-00209

**Published:** 2021-06-02

**Authors:** Tristan B. Weir, Tina Zhang, Julio J. Jauregui, Ali Aneizi, Patrick M.J. Sajak, Matheus B. Schneider, Mohit N. Gilotra, Joshua M. Abzug, R. Frank Henn, Ngozi M. Akabudike

**Affiliations:** From the Department of Orthopaedics, University of Maryland School of Medicine, Baltimore, Maryland.

## Abstract

We sought to determine whether patient and surgical factors are associated with the Press Ganey Ambulatory Surgery Survey (PGAS) satisfaction scores in patients undergoing outpatient upper extremity procedures.

**Methods:**

A retrospective review of a single academic urban hospital's Press Ganey database was performed for patients undergoing upper extremity procedures. PGAS scores above an a priori threshold were considered satisfied. Logistic regression analyses for the PGAS Total and Provider Scores were performed to determine the predictors of patient satisfaction.

**Results:**

Of the 198 patients included, the mean age was 49.6 ± 17.1 years and 55% were men. For the Total Score, multivariable analysis showed significantly less satisfaction with continuous catheter peripheral brachial plexus nerve blocks (CC-PNBs) (odds ratio [OR], 0.37; *P* = 0.008) and internet surveys (OR, 0.39; *P* = 0.007), but smokers had surprisingly more satisfaction (OR, 4.90; *P* = 0.016). For the Provider Score, a multivariable analysis showed less satisfaction with CC-PNBs (OR, 0.45; *P* = 0.035), internet surveys (OR, 0.46; *P* = 0.026), and geographic location (OR, 0.40; *P* = 0.005). Preoperative Patient-Reported Outcomes Measurement Information System scores were not associated with the PGAS scores.

**Discussion:**

Factors influencing satisfaction in patients undergoing upper extremity procedures may be modifiable (CC-PNBs and survey administration method) or nonmodifiable (geographic location) and may influence future reimbursement.

As the value of medical care becomes increasingly important in the United States, understanding the factors that influence the quality of care and patient experience is essential.^1^ The Centers for Medicare and Medicaid Services (CMS) established the Hospital Value-Based Purchasing Program to adjust hospital payments based on the quality of care provided.^2,3^ Although reporting of the patient experience is currently voluntary in the ambulatory surgery setting, practices may still use satisfaction data to adjust physician compensation and to improve patient care in anticipation of similar outpatient CMS initiatives.^4,5^

Patient satisfaction has been studied in multiple orthopaedic settings, including the orthopaedic hand clinic setting.^6,7^ Factors associated with patient satisfaction in hand clinic patients include age, wait time, whether an injection was performed, whether surgery was scheduled, and multiple domains of the Patient-Reported Outcomes Measurement Information System (PROMIS) outcomes measure.^7,8^ Patient satisfaction has also been a topic of interest in the total joint arthroplasty and spine surgery literature.^9,10,11,12^ However, we are unaware of any study assessing patient satisfaction in patients undergoing upper extremity procedures in the ambulatory surgery or hospital-based outpatient department setting. Having a more thorough understanding of the factors that influence upper extremity patients undergoing outpatient procedures will inform appropriate patient-mix adjustments and improve patient care.

The purpose of this study was to determine whether patient factors and preoperative PROMIS scores are associated with patient satisfaction in patients undergoing outpatient upper extremity procedures. We hypothesized that no patient or surgical factors would be associated with patient satisfaction for outpatient upper extremity procedures.

## Methods

After obtaining institutional review board approval, we queried our Press Ganey database for all patients who underwent an outpatient upper extremity procedure at a single hospital-based outpatient setting between July 2015 and August 2019. Of the 479 patients who underwent an outpatient upper extremity procedure and completed a Press Ganey satisfaction survey, 198 adult (18 years or older) patients were also enrolled in the Maryland Orthopaedic Registry (MOR) and included as the final cohort.^13^

Press Ganey is the largest CMS-approved survey vendor for patient satisfaction surveys, including the Press Ganey Ambulatory Surgery Survey (PGAS; Press Ganey Associates LLC, Elkridge, MD), which allows for public reporting of ambulatory and outpatient surgery satisfaction data.^14^ The PGAS survey contains 35 questions across six domains, including registration at the hospital, hospital facilities, nursing staff, care provider, personal issues, and the overall assessment of care.^14^ Questions use a Likert scale with answer choices ranging from “very poor” to “very good” (Appendix, http://links.lww.com/JG9/A135). The five response options are converted to scaled scores ranging from 0 (very poor) to 100 (very good).^14^ The mean scores for questions within a domain make up the domain subscore, and subscores are averaged to calculate the PGAS Total Score. A score of 100 for each domain subscore and for the Total Score indicates perfect satisfaction. Exclusion criteria include newborns, prisoners, publicity patients, patients admitted as inpatients, and patients who had a primary psychiatric diagnosis. Patients in the study completed a single survey. Additional datapoints obtained from the Press Ganey database included the patient's zip code to approximate the distance patients traveled to the hospital and whether the PGAS survey was administered via paper or internet.^4^

For the PGAS Total Score and Provider Subscore, patients were considered more satisfied if the mean score was above the 33rd percentile, whereas less satisfied patients had scores below this threshold. This a priori threshold was selected based on previous literature^4,7,8^ and corresponded with a mean Total Score of 88.5 points and a mean Provider Subscore of 93.8 points.

The MOR is a prospective web-based registry using the Research Electronic Data Capture data collection system.^13^ The Press Ganey database was linked to the MOR to provide potential predictors of PGAS scores. Patients were excluded from the MOR database if they were younger than 18 years old, not English-speaking, or incarcerated or a ward of the state. Variables obtained from the database included baseline demographic characteristics, body mass index, the American Society of Anesthesiology score, Charlson Comorbidity Index, smoking status, insurance type, education, employment status, income, procedure, type of anesthesia, and preoperative PROMIS computer adaptive test scores in six domains (Physical Function, Pain Interference, Fatigue, Social Satisfaction, Anxiety, and Depression). Anesthesia types included general, regional, or local. Regional anesthesia was stratified as continuous catheter (CC-PNB) or single-shot (SS-PNB) peripheral brachial plexus nerve blocks (PNB). Current Procedural Terminology codes were used to stratify procedure types. Procedures for trauma were defined as any procedure for a fracture or dislocation.

Continuous data were presented as the mean and SD, whereas categorical variables were presented as counts and percentages. Univariable logistic regression analyses were performed for the PGAS Total Score and Provider Subscore to predict the odds of patient satisfaction based on patient and surgical factors. Multivariable logistic regression models were created using a backward stepwise selection procedure for Total and Provider Score satisfaction. The selection procedure was set to remove factors at a *P* ≤ 0.20 threshold, starting with all variables in the univariable logistic regression with a *P* ≤ 0.20. All statistical analyses were performed using SPSS version 25.0 (IBM, Armonk, NY). Differences with *P* ≤ 0.05 were considered statistically significant.

## Results

Of the 479 patients who completed PGAS surveys during the study period, 198 patients were also enrolled in the MOR and were included in the final analysis. Patients had a mean age of 49.6 ± 17.1 years, 55% of the patients were men, and 72% were of White race (Table 1). The mean body mass index was 30.0 ± 7.9 kg/m^2^, and patients had relatively low mean Charlson Comorbidity Index (1.4 ± 1.3). A high proportion of patients had private or employer-based insurance (72%), a college degree or higher (69%), current employment or student (70%), and filled out an internet-based PGAS survey (62%; paper survey, 38%). Approximately half the patients earned more than $70,000 USD (49%) and lived within 15 miles of the hospital (44%). A slightly lower proportion of patients underwent surgery distal to the elbow (42%). Most patients underwent a PNB (84%), whereas lower proportions had general anesthesia (35%) or local anesthesia only (15%). Procedures for trauma made up 22% of the performed surgical procedures.

**Table 1:** Baseline Patient and Surgical Characteristics

Factors	Data^a^ (N = 198)
Patient characteristics	
Age (yr)	49.6 ± 17.1
Sex (male)	109 (55)
Race (White)	142 (72)
BMI, kg/m^2^	30.0 ± 7.9
ASA score	
1	52 (26)
2	115 (58)
3	31 (16)
CCI	1.4 ± 1.3
Current smoker	25 (13)
Insurance	
Medicare/Medicaid	54 (27)
Private/employer	142 (72)
Other	2 (1)
Education (college degree)	136 (69)
Employed or student	139 (70)
Income (≥$70 k)	96 (49)
Home within 15 miles	88 (44)
Internet survey	122 (62)
Surgical characteristics	
Anatomic location	
Shoulder/arm/elbow	115 (58)
Hand/wrist/forearm	83 (42)
Anesthesia	
General	69 (35)
CC-PNB	43 (22)
SS-PNB	123 (62)
Local	29 (15)
Procedure	
Hand (degenerative)	9 (5)
Neuroplasty	26 (13)
Hand tendon/ligament	23 (11)
Elbow tendon/ligament	9 (5)
Shoulder coracoid transfer/bone block	8 (4)
Shoulder arthroscopy	68 (34)
Trauma (below elbow)	29 (15)
Trauma (above elbow)	13 (7)
Arthroplasty	10 (5)
Other^b^	3 (1)

ASA = American Society of Anesthesiologists, BMI = body mass index, CCI = Charlson Comorbidity Index, CC-PNB = continuous catheter peripheral brachial plexus nerve block, SS-PNB = single-shot peripheral brachial plexus nerve block

a Values are given as the mean plus or minus the SD or as the number with the percentage in parentheses.

b Other procedures include implant removal (n = 2) and flap elevation (n = 1).

The univariable logistic regression for predictors of the PGAS Total Score showed current smokers, internet-based PGAS surveys, and receiving a CC-PNB were independent predictors of the Total Score (Table 2). These predictors remained notable in the multivariable logistic regression model (Table 3). Current smokers were nearly five times as likely to be satisfied (odds ratio [OR], 4.90; 95% confidence interval [CI], 1.35 to 17.67; *P* = 0.016), whereas patients completing internet-based surveys were 61% less satisfied (OR, 0.39; 95% CI, 0.20 to 0.78; *P* = 0.007) and patients who received a CC-PNB were 63% less satisfied (OR, 0.37; 95% CI 0.18 to 0.78; *P* = 0.008). Living within 15 miles of the hospital trended toward less satisfaction, but this finding was not statistically significant (OR, 0.53; 95% CI, 0.28 to 1.01; *P* = 0.054).

**Table 2:** Univariable Logistic Analysis for the PGAS Total and Provider Score

Factors	Total Score^a^		Provider Score^a^	
	OR (95% CI)	*P*	OR (95% CI)	*P*
Patient characteristics				
Age, per year	0.99 (0.98-1.02)	0.92	1.00 (0.99-1.02)	0.65
Male sex, ref. female	0.59 (0.32-1.08)	0.087	0.70 (0.38-1.29)	0.25
Non-White, ref. no	1.21 (0.62-2.35)	0.58	0.72 (0.38-1.39)	0.33
BMI, per kg/m^2^	1.02 (0.98-1.06)	0.40	1.01 (0.97-1.05)	0.61
ASA score, per point	0.95 (0.60-1.50)	0.81	1.20 (0.75-1.92)	0.45
CCI, per point	1.00 (0.80-1.25)	1.00	1.00 (0.80-1.26)	0.98
Current smoker, ref. no	4.20 (1.21-14.59)	0.024^b^	1.60 (0.61-4.22)	0.34
Private insurance, ref. no	0.83 (0.43-1.61)	0.58	0.78 (0.40-1.54)	0.48
College degree, ref. no	0.53 (0.27-1.05)	0.068	0.50 (0.25-1.00)	0.05^b^
Employed, ref. no	0.59 (0.30-1.16)	0.13	0.55 (0.28-1.11)	0.094
Income ≥$70k, ref. no	0.83 (0.46-1.51)	0.55	0.91 (0.50-1.66)	0.77
Home ≤15 mi., ref. no	0.59 (0.33-1.08)	0.087	0.41 (0.22-0.75)	0.004^b^
Internet survey, ref. paper	0.43 (0.22-0.82)	0.011^b^	0.46 (0.24-0.88)	0.019^b^
Surgical characteristics				
Elbow and distal, ref. proximal	1.29 (0.70-2.35)	0.42	1.31 (0.71-2.41)	0.38
Trauma, ref. no	0.77 (0.38-1.56)	0.46	1.08 (0.52-2.26)	0.83
Soft tissue, ref. bone	0.74 (0.40-1.37)	0.34	1.03 (0.55-1.93)	0.92
Anesthesia				
General, ref. no	0.91 (0.49-1.68)	0.75	1.14 (0.61-2.15)	0.68
CC-PNB, ref. no	0.38 (0.19-0.76)	0.006^b^	0.46 (0.23-0.91)	0.026^b^
SS-PNB, ref. no	1.61 (0.88-2.95)	0.12	1.75 (0.95-3.21)	0.073
Local, ref. no	1.13 (0.48-2.64)	0.78	0.89 (0.39-2.05)	0.79

ASA = American Society of Anesthesiologists, BMI = body mass index, CI = confidence interval, CCI = Charlson Comorbidity Index, CC-PNB = continuous catheter peripheral brachial plexus nerve block, OR = odds ratio, PGAS = Press Ganey Ambulatory Surgery Survey, SS-PNB = single-shot peripheral brachial plexus nerve block

a Patients with scores over the 33rd percentile were considered more satisfied, whereas those below the 33^rd^ percentile were considered less satisfied.

b Indicates statistically significant values (*P* ≤ 0.05).

**Table 3:** Multivariable Logistic Analysis for the PGAS Total and Provider Score

Factors	Total Score^a^		Provider Score^a^	
	OR (95% CI)	*P*	OR (95% CI)	*P*
CC-PNB, ref. no	0.37 (0.18-0.78)	0.008^b^	0.45 (0.22-0.95)	0.035^b^
Internet survey, ref. paper	0.39 (0.20-0.78)	0.007^b^	0.46 (0.24-0.91)	0.026^b^
Home ≤15 mi., ref. no	0.53 (0.28-1.01)	0.054	0.40 (0.21-0.76)	0.005^b^
Current smoker, ref. no	4.90 (1.35-17.67)	0.016^b^	—	—
College degree, ref. no	—	—	0.55 (0.27-1.14)	0.11

CI = confidence interval, CC-PNB = continuous catheter peripheral brachial plexus nerve block, OR = odds ratio, PGAS = Press Ganey Ambulatory Surgery Survey

a Patients with scores over the 33rd percentile were considered more satisfied, whereas those below the 33^rd^ percentile were considered less satisfied.

b Indicates statistically significant values (*P* ≤ 0.05).

The univariable logistic regression for predictors of the PGAS Provider Score showed that obtaining a college degree, living within 15 miles of the hospital, completing an internet-based PGAS survey, and receiving a CC-PNB were independent predictors of the Provider Score (Table 2). All variables, except for obtaining a college degree, remained notable in the multivariable logistic regression model (Table 3). Patients who received a CC-PNB were 55% less satisfied with their provider (OR, 0.45; 95% CI 0.22 to 0.95; *P* = 0.035), those who completed an internet survey were 54% less satisfied (OR, 0.46; 95% CI 0.24 to 0.91; *P* = 0.026), and those living within 15 miles of the hospital were 60% less satisfied (OR, 0.40; 95% CI 0.21 to 0.76; *P* = 0.005). Patients who had a college degree trended toward less satisfaction but were not statistically significant (OR, 0.55; 95% CI 0.27 to 1.14; *P* = 0.11).

None of the six PROMIS domains were significantly associated with the PGAS Total Score on univariable or multivariable logistic regression analyses (*P* > 0.05; Table 4).

**Table 4:** Logistic Regression for PGAS Total Score Based on PROMIS

PROMIS CAT	Univariable^a^	Multivariable^a,b^	*P* ^c^
	OR (95% CI)	OR (95% CI)	
Physical function	0.98 (0.95-1.01)	0.99 (0.95-1.02)	0.49
Pain interference	1.00 (0.96-1.04)	0.99 (0.94-1.04)	0.68
Fatigue	0.99 (0.97-1.03)	0.99 (0.96-1.02)	0.65
Social satisfaction	0.99 (0.96-1.02)	0.99 (0.97-1.03)	0.95
Anxiety	1.00 (0.96-1.03)	0.99 (0.96-1.03)	0.69
Depression	0.99 (0.96-1.02)	0.99 (0.96-1.02)	0.43

CAT = computer adaptive test, CI = confidence interval, OR = odds ratio, PGAS = Press Ganey Ambulatory Surgery Survey, PROMIS = Patient-Reported Outcomes Measurement Information System

a Patients with scores over the 33rd percentile were considered more satisfied, whereas those below the 33rd percentile were considered less satisfied.

b Additional variables included in the model but not shown are home within 15 miles, internet survey, smoking, interscalene catheter, surgeon.

c *P* value presented for the multivariable logistic regression analysis.

## Discussion

Because CMS continues to expand the Hospital Value-Based Purchasing Program, patient satisfaction in the outpatient surgical setting will gain more importance for practices to optimize incentives and improve patient care. Although previous studies have focused on the factors influencing patient satisfaction in the hand clinic setting,^7,8^ ours is the first to evaluate similar factors in patients undergoing upper extremity procedures in the ambulatory surgical setting. The results of this study show patient, surgical, and survey factors markedly influence the Total and Provider Scores in patients undergoing upper extremity procedures. This may help practices recognize potential risk factors for patient dissatisfaction, help providers set appropriate expectations for surgery, and provide policymakers with data to make appropriate patient-mix adjustments.

CC-PNBs have been commonly used to provide postoperative analgesia for a variety of upper extremity surgical procedures;^15^ however, our results show that patients with continuous catheters reported lower PGAS Total and Provider Scores. Continuous catheters can reduce the early rebound pain seen with traditional SS-PNBs but have multiple potential drawbacks including infusion pump failure, catheter displacement, and block failure because of technical errors.^15,16^ They also require patients to maintain the infusion pump, remove the catheter when the pump is empty, and potentially return the device to the manufacturer. It is possible that such inconveniences and implications on postoperative pain could have contributed to the 63% reduction in the PGAS Total Score observed in this study. Patients with continuous catheters also had 55% lower odds of being satisfied with the surgeon. This is consistent with previous literature showing that patients associate pain management with hospital satisfaction in other areas of orthopaedics.^17,18^ Recent advances with SS-PNBs seek to improve the drawbacks of continuous catheters by using long-acting compounds,^19-21^ but improved patient satisfaction with SS-PNBs only trended toward significance in our univariate models for the Total Score (OR, 1.61; 95% CI, 0.88 to 2.95) and Provider Score (OR, 1.75; 95% CI 0.95 to 3.21). Further studies should assess whether specific SS-PNBs agents provide improved patient satisfaction but was beyond the scope of this study. Our results suggest CC-PNBs are modifiable factors that are associated with lower patient satisfaction scores, but further studies should determine the reasons for this association.

Although PGAS survey administration and public reporting is currently voluntary, outpatient surgery centers are currently using this survey in preparation for CMS mandates in the outpatient setting. Important to public reporting and possible future incentives is the patient-mix adjustment, which adjusts scores based on patient demographics, hospital characteristics, and other factors. Such factors include the mode of survey administration because patients tend to be more satisfied when surveys are completed over the telephone.^14^ Our results show patients who complete internet surveys have 61% and 54% lower odds of being satisfied based on the Total and Provider Scores, respectively. This contrasts with previous findings in spine surgery clinic patients, where online survey completion was associated with improved satisfaction when compared with paper surveys.^22^ Although not statistically significant, we also showed a trend toward lower satisfaction in more educated and employed patients. This could potentially indicate that patients with higher socioeconomic status are more critical of the medical care they receive. We suspect that such differences in socioeconomic status could account for differences in satisfaction based on access to fill out an internet-based survey, but this requires further study. Consistent with previous literature in orthopaedic clinic patients,^4^ we also showed patients living closer to the hospital have lower satisfaction. Although the living distance from the hospital could be an indicator of socioeconomic status, notable differences only existed for non-White race (20% greater versus 39% less than 15 miles; *P* = 0.004) but not employment status, income, and insurance status. This could indicate that patients who rate the hospital and provider more favorably are more willing to travel a greater distance to undergo surgery. Such nonmodifiable factors should be considered when making patient-mix adjustments and reporting these results to the public.

In upper extremity clinic patients, Tyser et al^8^ showed that the physical function, anxiety, and pain interference PROMIS domains were markedly associated with Press Ganey satisfaction scores. This contrasts with our results because we did not show a correlation between PROMIS domains and patient satisfaction. These discrepancies could be related to differences in the clinic versus surgical setting or differences in the Press Ganey questionnaires. However, given our odds ratios close to one with narrow confidence intervals, we do not believe that PROMIS is predictive of PGAS scores in upper extremity patients. Similarly, previous studies in orthopaedic literature have failed to show differences between Press Ganey survey results and patient-reported outcomes.^9,10^ With such inconsistent results between accepted patient-reported outcomes and Press Ganey satisfaction results, surgical practices and policymakers may question the utility of the PGAS survey's ability to capture meaningful outcomes.

Finally, we surprisingly showed that current smokers have higher PGAS Total Scores than nonsmokers. Smoking is generally regarded as a negative risk factor because of the vasoconstrictive effects of nicotine that reduce blood supply and disrupt healing.^23^ In addition, smoking can have negative effects on upper extremity-specific outcomes,^24^ and previous studies have shown that smokers have lower Press Ganey satisfaction results.^11,12^ Therefore, our finding that smokers have higher satisfaction may be because of chance and should be interpreted with caution. This finding further shows inconsistencies between Press Ganey surveys and well-accepted negative risk factors for patients undergoing orthopaedic procedures and questions the validity of such satisfaction surveys to capture meaningful data.

Multiple limitations were noted in this study. First, the study was retrospective in nature and includes a heterogenous group of patients undergoing various types of upper extremity surgery. Although it would be beneficial to stratify patients according to specific procedures, we were not powered to do so. However, PGAS survey results are reported in a heterogenous manner and do not stratify by procedure, making our results more relatable to hospitals and surgeons. Second, Press Ganey surveys are subject to particularly high rates of nonresponse bias. Typical response rates are between 15 to 20%,^10^ but one study reported a rate of 8.9%.^7^ In addition, only 41% of the patients who underwent an upper extremity procedure and completed a PGAS survey were also enrolled in the MOR, introducing additional potential selection bias. We did, however, confirm that no notable differences were observed in PGAS scores between patients who were and were not enrolled in the MOR. We chose to only include patients concurrently enrolled in the MOR so we could assess more variables of interest, including PROMIS scores, than those limited to the PGAS survey alone. Finally, this study represents data from a single academic outpatient surgery setting in an urban location, which could limit the generalization of the findings.

In summary, this is the first study to evaluate the PGAS survey in patients undergoing upper extremity procedures. We showed patients who receive a CC-PNB, complete an internet survey, or live within 15 miles of the hospital have lower satisfaction scores. There was a trend toward lower satisfaction in more educated patients, whereas smokers had surprisingly higher satisfaction scores. PROMIS scores were not predictive of satisfaction in patients undergoing upper extremity procedures. These results should be used to create appropriate patient-mix adjustments for hospital reporting and provides information about modifiable and nonmodifiable risk factors for dissatisfaction.
